# Exploring the Interactions of Oncolytic Viral Therapy and Immunotherapy of Anti-CTLA-4 for Malignant Melanoma Mice Model

**DOI:** 10.3390/cells12030507

**Published:** 2023-02-03

**Authors:** Jui-Ling Yu, Sophia R.-J. Jang, Kwei-Yan Liu

**Affiliations:** 1 Department of Data Science and Big Data Analytics, Providence University, Taichung City 43301, Taiwan; 2 Department of Mathematics and Statistics, Texas Tech University, Lubbock, TX 79409, USA; 3 National Institute of Environmental Health Sciences, National Health Research Institutes, Miaoli County 53053, Taiwan

**Keywords:** mathematical modeling, oncolytic virus therapy, immune checkpoint CTLA-4, cytokines, melanoma, 92D25, 92B05

## Abstract

Oncolytic ability to direct target and lyse tumor cells makes oncolytic virus therapy (OVT) a promising approach to treating cancer. Despite its therapeutic potential to stimulate anti-tumor immune responses, it also has immunosuppressive effects. The efficacy of OVTs as monotherapies can be enhanced by appropriate adjuvant therapy such as anti-CTLA-4. In this paper, we propose a mathematical model to explore the interactions of combined therapy of oncolytic viruses and a checkpoint inhibitor, anti-CTLA-4. The model incorporates both the susceptible and infected tumor populations, natural killer cell population, virus population, tumor-specific immune populations, virus-specific immune populations, tumor suppressive cytokine IFN-g, and the effect of immune checkpoint inhibitor CTLA-4. In particular, we distinguish the tumor-specific immune abilities of CD8^+^ T, NK cells, and CD4^+^ T cells and describe the destructive ability of cytokine on tumor cells as well as the inhibitory capacity of CTLA-4 on various components. Our model is validated through the experimental results. We also investigate various dosing strategies to improve treatment outcomes. Our study reveals that tumor killing rate by cytokines, cytokine decay rate, and tumor growth rate play important roles on both the OVT monotherapy and the combination therapy. Moreover, parameters related to CD8^+^ T cell killing have a large impact on treatment outcomes with OVT alone, whereas parameters associated with IFN-g strongly influence treatment responses for the combined therapy. We also found that virus killing by NK cells may halt the desired spread of OVs and enhance the probability of tumor escape during the treatment. Our study reveals that it is the activation of host anti-tumor immune system responses rather than its direct destruction of the tumor cells plays a major biological function of the combined therapy.

## 1. Introduction

Oncolytic virotherapy has shown promising anti-tumoral effects in numerous basic and clinical research. Besides the oncolytic ability to direct target and lyse tumor cells, oncolytic viruses (OVs) also can be designed to selectively replicate to kill tumor cells while preventing their binding to and replication in healthy normal cells through engineered mutations [1]. Nevertheless, infectious virus particles increase with time within the tumor, which may prevent patients’ exposing to substantial excess risk during drug delivery [2] and the tumor-selective replication of viruses makes cancer treatment less toxic than standard chemotherapy drugs [3]. Because OVs can be made in the laboratory by modifying their genome, this allows therapeutic exploitation of viruses encoded with transgene proteins to against T cell inhibitory factors such as anti-CTLA-4. Clinical evidence has showed that combining oncolytic viruses with checkpoint inhibitors CTLA-4 in various cancer types, including prostate, and melanoma xenografts, resulted in upregulation of intratumoral T cells and boosting T cell immunity to solid tumors [4,5]. These molecular biotechnology modifications harness the immune system to eliminate tumor cells and provide a new treatment avenue for durable and effective clinical responses in cancer patients [6].

An activation of CD8^+^ T cells requires two signals. The first signal occurs when a naive CD8^+^ T cell encounters and interacts with an antigen presenting cell such as a dendritic cell through the T cell receptor [7]. The second signal, the co-stimulation signal, is provided by the interaction between CD28 on the membrane of T cells and B7 on antigen presenting cells [7,8]. However, CTLA-4 (cytotoxic T lymphocyte antigen-4) molecules are expressed by activated T cells such as CD4^+^ T cells and regular T cells and can out-compete CD28 for binding to B7 and thus dampen the co-stimulatory signals. In 2011, the U.S. Food and Drug Administration approved ipilimumab, an anti-CTLA-4 antibody, for melanoma to increase the mobility of T cells and allow the cytotoxic T cells to continue to destroy cancer cells [9,10,11,12]. Unfortunately, not all patients responded and the long-term treatment efficacy was not satisfactory in solid tumors [13]. Combining the OVT with CTLA-4 blockade could complement the effects of poor tumor targeting through the selective replication ability of OVs in tumor cells and thus dramatically boost anti-tumor therapeutic efficacy [14].

There are several mathematical systems modeling the interactions between OVs and tumor cells along with various immune responses. For example, Mahasa et al. used a model of delay differential equations to describe interactions of normal and tumor cells by considering adaptive immune responses followed by an early viral propagation period. They assumed that OVs infect both host and cancer cell populations and subsequently induce anti-viral immune responses [15]. Eftimie et al. [16] built a mathematical model to study the anti-tumor effect of adenovirus and oncolytic vesicular stomatitis virus, and their interactions with the CD8^+^ T related immune cells. Their study indicated that cancer treatment could be improved with different types of oncolytic viruses. Senekal et al. built a mathematical model to investigate the dynamical interactions of OV-induced NK cell recruitment during oncolytic virotherapy. Their study suggested that NK response simulated by OV is more efficient at reducing the infected tumor cell population than stimulated by the presence of tumor only. They also suggested other long-term effector cells such as CD8^+^ T cells should be included in the OV model building [17]. Storey and Jackson developed a spatially explicit hybrid cellular automaton and partial differential equations with a combination of an oncolytic viral therapy and an anti-PD-1 immunotherapy to investigate the influence of spatial location of viral doses and to determine optimal viral dosing to therapeutic efficacy. Their results indicated that the tumor antigenicity level plays a more important role for treatment efficacy than the T cell killing rate [18]. Most of the oncolytic viral therapy models including checkpoint pathway address the discussion of the PD-1/PD-L1 checkpoint in which the immune responses are conveyed by anti-tumor CD8^+^ T cells.

Deviated from the above mentioned approaches, our model studies the effectiveness of oncolytic virotherapy together with immune checkpoint modulators CTLA-4. We include the effect of cytokines, which is seldom addressed in other models. Although there are more than ten types of immune cells that are known to play a vital role in oncolytic virotherapy, for simplicity, we only consider essential to therapeutic effect of OV such as CTL and NK cells which are addressed by immune cell depletion studies [19]. Moreover, our model construction is based on the population level interactions and does not account for subcellular events and stimulatory pathways. Since CTLA-4 molecules are expressed mostly by activated CD4^+^ T cells and little by CD8^+^ T cells, and CD4^+^ T cells are stimulated by cytokine IFN-γ, their influences are considered in the immune responses. The CTLA-4 is considered as a negative regulator of CD4^+^ T cell activation, and, as a consequence, can prevent cytokine production. Unlike CD4^+^ T cells, the anti-CTLA-4 does not have a significantly influence on the proliferation of CD8^+^ T cells. Chan et al. shows that checkpoint-blocking activity of anti-CTLA-4 on CD4^+^ T cells permits greater production in CD4^+^ T cells than in CD8^+^ T cells [20]. Therefore, the population of CD8^+^ T cells is not considered to be influenced by the CTLA-4 in our model. Specifically, we distinguish the tumor-specific immune abilities of CD8^+^ and CD4^+^ T cells: effector CD8^+^ T cells directly kill susceptible and infected tumor cells while CD4^+^ T cells kill tumor cells indirectly through cytokines. T cells are also recruited by immune cells, which become activated when they encounter viruses. The innate immune NK cell population is assumed to be pre-existing within the tumor vicinity and is recruited to the TME and mediates initial OV clearance as in [17]. They are activated due to immunogenic cell death of infected tumor cells and cytokines. Cytokines activated by both susceptible and infected tumor cells and secreted by activated T cells and NK cells conduct the indirect killing. In addition to the tumor-specific immune response, we also take into the account of virus-specific immune response due to the fact that the presence of virons on infected tumor cells also activates anti-viral immune responses. They serve to constrain viral infections by viral lysis free OVs. Our proposed model is supported by the murine experiment of Engeland et al. [5] in which the treatment of murine model of malignant melanoma B16-CD20 using OVT and the immune checkpoint inhibitor CTLA-4 are conducted [5].

The remainder of this paper is organized as follows: In Section 2, we describe our mathematical model incorporating the combined treatment of OVT and anti-CTLA-4. We describe in detail the assumptions considered for each equation in the full model derived, and we parameterize the model parameters. Numerical explorations are provided in Section 3. In particular, Section 3.1 presents model validation and Section 3.2 investigates various treatment protocols for improved therapeutic outcomes. Section 3.3 performs global sensitivity analysis using either the monotherapy of OVT or the adjuvant therapy with anti-CTLA4. The Section 4 presents a brief review and discussion.

## 2. Model Construction and Parameterization

### 2.1. Model Development

**Figure 1:**
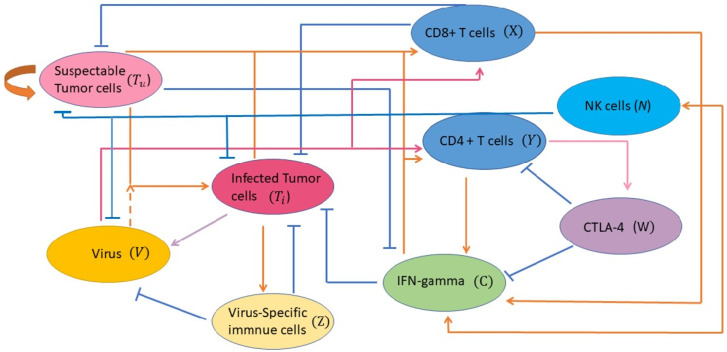
A schematic diagram depicts the interaction between oncolytic virus, immune cells, cytokines, and immune checkpoint with tumor cells. Uninfected tumor cells become infected by an oncolytic virus is presented. After successful viral penetration within the infected cells, infected cancer cells lyse and produce new infectious viral particles. Fragments from infected cancer cells stimulate anti-viral immune cells which subsequently kill infected cells and clear free virus. The anti-tumor immune cells (CD8^+^ T, NK cells, and CD4^+^ T cells) attack and destroy (direct/indirectly) both infected and uninfected cancer cells. T cells are also recruited by innate NK immune cells which become activated when they encounter the viruses. Cytokines activated by both susceptible and infected tumor cells, and secreted by activated T cells and NK cells, conduct the indirectly killing via activating macrophages, increasing phagocytosis of pathogen and tumor cells. Soluble proteins CTLA-4 are expressed by the activated CD4^+^ T cells and CD8^+^ T cells. It acts as a negative regulator of CD4^+^ T cell activation and prevents cytokine productions. NK cells are activated due to immunogenic cell death of infected tumor cells, leading to recruitment of NK cells. They are also activated in response to cytokines and vice versa. Finally, NK cells clear free viruses.

We formulate a simple ODE model to investigate the treatment of murine model of malignant melanoma B16-CD2 using OVT and anti-CTLA-4 [5]. The goals of the proposed model are to predict: combining oncolytic virotherapy with immune checkpoint modulators would reduce tumor burden by direct cell lysis and stimulating anti-tumor immunity as described in [5]. The model is constructed to describe the interactions between tumor populations, virus population, tumor-specific CTL response, NK cells, virus-specific immune populations, tumor suppressive cytokine IFN-γ, and the effect of immune checkpoint inhibitor CTLA-4. We consider separately the average temporal changes in the uninfected tumor size ($T_{u}\left(t\right)$) and infected tumor size ($T_{i}\left(t\right)$). For the immune response, we model the evolution of effector cells CD8^+^ T (X(t)) and CD4^+^ T (Y(t)) cells, the population of co-inhibitory receptor CTL-associated antigen 4 (CTLA-4) molecules (W(t)) expressed by the CD4^+^ T cells and CD8^+^ T cells. The specific or non-specific attack of NK cells during oncolytic virotherapy is specified by the variable (N(t)). To model the evolution of CD4^+^ T cells, we focus on the effector role of CD4^+^ T cells. In fact, CD4^+^ T cells can kill cancer cells through cytokines and chemokines they produced even in the absence of CD8^+^ T cells and NK cells [21,22,23,24]. Since the level of tumor suppressive IFN-γ is investigated in mice treated with OVs and aCTLA-4 in [5] and tumor eradication or recrudesced after initial regression was found in mice lacking of IFN-γ recipient [25], the effect of IFN-γ (C(t)) is considered within the model. We model the virus population (V(t)) and the total number of anti-viral immune cells (Z(t)) to describe the immune responses to viral infection. The time unit is a day. The cell population has the unit of number of cells while oncolytic virions have the unit of PFU. The cytokines and immune checkpoint have the units of pg/mL and number of molecules, respectively. Prior to the model introduction, we describe the assumptions considered for each equation in the full model. These assumptions are depicted schematically in Figure 1. The summary of model state variables with their respective definitions are presented in Table 1.

- Equation (1) models the uninfected tumor cell population. We assume susceptible tumor cells grow in a logistic fashion with an intrinsic growth rate $r_{u}$ and carrying capacity $K_{t}$ for all tumor cells. We choose logistic growth model for the susceptible tumor population because evidence shows that the growth of tumor is slower when the tumor becomes larger [15,26]. The term $\beta_{t}\frac{T_{u}V}{m_{v}+T_{u}}$ is the viral infection rate of uninfected tumor cells with the maximum rate $\beta_{t}$, which turns a susceptible tumor cell into an infected tumor cell. The saturated form of the tumor–virus interaction is considered according to [26]. The killings of tumor cells are modeled by the terms $\delta_{x}\frac{X}{m_{x}+X}T_{u}$ and $\delta_{c}\frac{T_{u}}{m_{t}+T_{u}}C$ due to CD8^+^ T cells and cytokines, respectively. They represent susceptible tumor population killed directly by the effector CD8^+^ T cells and indirectly by CD4^+^ T cells through cytokines at a rate of $\delta_{x}$ and $\delta_{c}$, respectively. The Michaelis–Menten kinetics form of the tumor and the tumor-specific immune cell interaction is adopted according to [15] and [27]. The saturated form shows the limited property of effector cells and cytokines abilities to lyse tumor cells. NK cells are able to recognize tumor antigens and indiscriminately kill tumor cells. The term $d_{u}NT_{u}$ is used to model the tumor killing by NK cells at a rate $d_{u}$.
- Equation (2) models the population of infected tumor cells. Similar to susceptible tumor cells, we assume infected tumor cells grow in a logistic fashion with an intrinsic growth rate $r_{i}$ and carrying capacity $K_{t}$ for all tumor cells. Susceptible tumor cells infected by oncolytic virus at a rate $\beta_{t}$ result in the increase of the infected tumor cells. This cell population dies at a rate $a_{t}$. Since T cells kill not only virus-free tumor cells but also virus-infected tumor cells, the infected tumor cells are lysed via anti-tumor adaptive immune cells CD8^+^ T cells (directly killing) with $\delta_{x}\frac{X}{m_{x}+X}T_{i}$ and by CD4^+^ T cells (indirectly killing through cytokines) with $\delta_{c}\frac{T_{i}}{m_{t}+T_{i}}C$. The killing due to anti-viral adaptive immune cells is modeled by the term $\delta_{z}ZT_{i}$ at a rate $\delta_{z}$ [15]. The term $d_{i}NT_{i}$ is used to address the killing by NK cells on infected tumor cells at a constant rate $d_{i}$.
- Equation (3) models the virus population. New virus particles are produced upon clearance of an infected cancer cell. The parameter $b_{t}$ is the burst size of viruses released from an infected tumor cell. $\delta_{v}$ is the viral lysis by anti-viral immune cells. The virus decays at a rate $\gamma_{v}$ and *s* represents the dose of OV. NK cells not only directly recognize and kill viral-infected cells through their receptors but also provide an antigen-specific adaptive response to viral infections, which represents the first line of defense and a rapid immune response against viral infections [28]. The term $d_{v}NV$ models virus killing of free OV by NK cells at a rate $d_{v}$.
- Equation (4) models tumor-specific CD8^+^ T cells related adaptive immune responses. The term $a_{x}\frac{T_{i}+T_{u}}{h_{x}+T_{i}+T_{u}}X$ models proliferation of CD8^+^ T cells. Here, a Michaelis–Menten term is used to denote that the anti-tumor immune response is induced by tumor antigens presented on both uninfected and infected tumor cells [15], where $h_{x}$ is the half-saturation constant and $a_{x}$ is the proliferation rate of anti-tumor adaptive immune cells. Since anti-tumor immune response is activated by oncolytic viruses to fight tumor cells, the term $a_{v}NV$ denotes the rate for which T cells are recruited by immune cells through the interactions with viruses at a rate $a_{v}$. This addresses the T cell activation following encounter with the viruses, rather than by encountering with infected cells [29]. $\gamma_{x}$ is the natural death rate of anti-tumor T cells.
- Equation (5) models tumor-specific CD4^+^ T cells related adaptive immune responses (e.g., cytokine related immune responses). The evolution of CD4^+^ T cells takes similar proliferation formation as that of Equation (4) but with different half-saturation constant $h_{y}$. Since CD4^+^ T cells are released mainly by IFN-γ and anti-CTLA-4 amplifies CD4^+^ T cell activation [27], the proliferation of CD4^+^ T is modeled in the form of $a_{y}\frac{T_{i}+T_{u}}{\left(h_{y}+T_{i}+T_{u}\right)\left(1+\nu W\right)}C$, where $a_{y}$ is the proliferation rate of Th cells. The inhibition of CTLA-4 on CD4^+^ T cells is modeled by the term (1+νW) with a measure of inhibition ν. Parameter $\gamma_{y}$ is the apoptosis rate of Th cells.
- Equation (6) models the time evolution of major cytokine IFN-γ. T cells and NK cells produce several cytokines and chemokines that coordinate various immune responses and are the major source of IFN-γ. The terms $\alpha_{x}\frac{\left(T_{i}+T_{u}\right)}{1+b_{x}W}X$, $\alpha_{y}\frac{\left(T_{i}+T_{u}\right)}{1+b_{y}W}Y$, and $\alpha_{n}\frac{\left(T_{i}+T_{u}\right)}{1+b_{n}W}N$ represent that IFN-γ is activated by both susceptible and infected tumor cells, and secreted by activated CD8^+^ T [20,30], CD4^+^ T cells [20,25], and NK cells [31] at a constant rate $\alpha_{x}$, $\alpha_{y}$, and $\alpha_{n}$, respectively. The terms $1+b_{x}W$, $1+b_{y}W$, and $1+b_{n}W$ model CTLA-4 engagement that prevents cytokine production and the fact that anti-CTLA-4 therapy results a significant amount of IFN-γ [25,32]. The parameter $\gamma_{c}$ is the natural degradation rate of IFN-γ.
- Equation (7) models soluble proteins CTLA-4. The CTLA-4 molecules are expressed on activated T cells such as effector T cells and regulatory T cells and can out-compete CD28 for binding to B7 and thus dampen the co-stimulatory signals. The CTLA-4 expression rate on a single CD4^+^ T cell is assumed a constant and is denoted by the parameter $r_{y}$. Evidence has shown that CTLA-4 is also expressed on CD8^+^ T cells [20]. The CTLA-4 expression rate on a single CD8^+^ T cell is denoted by the parameter $r_{x}$. The natural lost rate of CTLA-4 is denoted by $\gamma_{w}$. The blockade rate of immune checkpoint CTLA-4 is presented by the parameter *u*.
- Equation (8) models the virus-specific immune response. The equation describes immune responses to viral infection. The parameter $p_{v}$ is the virus-specific proliferate rate of anti-viral immune cells which become activated due to debris or viral antigens on infected cells [15]. The natural death rate of anti-viral immune cells is denoted by $\gamma_{z}$.
- Equation (9) models natural killer (NK) cells. NK cells are known to be non-specific and attack “non-self” cells, but NK cells can be specific or non-specific during oncolytic virotherapy [17]. There is a pre-existing NK cell population ($s_{N}$) within the tumor vicinity and is recruited to the TME and mediates initial OV clearance [17]. In this study, we assume activation of NK cells is dependent on the contact with OV infected tumor cells, as experimentally observed in Leung et al. [33]. The second term, $r_{n}N\left(1-\frac{N}{K_{n}}\right)\left(\frac{T_{i}}{T_{i}+m_{n}}\right)$, represents the stimulation and recruitment of NK cells. A saturation term $\frac{T_{i}}{T_{i}+m_{n}}$ is used to describe the limited effects of NK response by infected tumor cells. We also assume that NK cell response is further enhanced by lysis, which induces immunogenic cell death (ICD) of infected tumor cells at the rate $r_{n}$ until it attains the maximum capacity $K_{n}$. Parameter $r_{n}$ is the recruitment/proliferation of NK cells in response to danger signals (such as DAMPs and PAMPs) released during ICD of OV-infected tumor cells. Parameter $m_{n}$ represents the half-saturation constant of NK cells that supports the maximum killing of tumor cells by NK cells. Proliferation of NK cells is also stimulated by IFN-γ [34]. Parameter $h_{n}$ is used to indicate the saturated effects of immune response, and parameter ζ is the stimulation and recruitment of NK cells by IFN-γ. The interactions of NK cells with tumor cells can lead to inactivation of NK cells at a constant rate, $\delta_{n}$ that is proportional to their interaction term. In fact, $-\delta_{n}\left(T_{u}+T_{i}\right)N$ denotes the inactivation of NK cells upon their interactions with tumor cells at the rate $\delta_{n}$. Finally, the last term, $\gamma_{n}N$, denotes the natural death rate of NK cells. The formulation of equation of NK cells is derived from [17,34].

The detailed description of model parameters is summarized in Table 2, and the model is given in Equations (1)–(10).

(1) $$\begin{array}{ccc} \frac{dT_{u}}{dt} & = & r_{u}T_{u}\left(1-\frac{T_{u}+T_{i}}{K_{t}}\right)-\beta_{t}\frac{T_{u}}{m_{v}+T_{u}}V-\delta_{c}\frac{T_{u}}{m_{t}+T_{u}}C-\delta_{x}\frac{X}{m_{x}+X}T_{u}-d_{u}NT_{u} \end{array}$$

(2) $$\begin{array}{ccc} \frac{dT_{i}}{dt} & = & r_{i}T_{i}\left(1-\frac{T_{u}+T_{i}}{K_{t}}\right)+\beta_{t}\frac{T_{u}}{T_{u}+m_{v}}V-a_{t}T_{i}-\delta_{x}\frac{X}{m_{x}+X}T_{i}-\delta_{c}\frac{T_{i}}{m_{t}+T_{i}}C\\ & - & \delta_{z}ZT_{i}-d_{i}NT_{i} \end{array}$$

(3) $$\begin{array}{ccc} \frac{dV}{dt} & = & b_{t}a_{t}T_{i}-\delta_{v}VZ-\gamma_{v}V-d_{v}NV+s \end{array}$$

(4) $$\begin{array}{ccc} \frac{dX}{dt} & = & a_{v}NV+a_{x}\frac{T_{i}+T_{u}}{h_{x}+T_{i}+T_{u}}-\gamma_{x}X \end{array}$$

(5) $$\begin{array}{ccc} \frac{dY}{dt} & = & \frac{a_{v}NV}{1+\nu W}+a_{y}\frac{T_{i}+T_{u}}{\left(h_{y}+T_{i}+T_{u}\right)\left(1+\nu W\right)}C-\gamma_{y}Y \end{array}$$

(6) $$\begin{array}{ccc} \frac{dC}{dt} & = & \alpha_{n}\frac{\left(T_{i}+T_{u}\right)}{1+b_{n}W}N+\alpha_{x}\frac{\left(T_{i}+T_{u}\right)}{1+b_{x}W}X+\alpha_{y}\frac{\left(T_{i}+T_{u}\right)}{1+b_{y}W}Y-\gamma_{c}C \end{array}$$

(7) $$\begin{array}{ccc} \frac{dW}{dt} & = & r_{x}\left(1-u\right)X+r_{y}\left(1-u\right)Y-\gamma_{w}W \end{array}$$

(8) $$\begin{array}{ccc} \frac{dZ}{dt} & = & p_{v}T_{i}-\gamma_{z}Z \end{array}$$

(9) $$\begin{array}{ccc} \frac{dN}{dt} & = & s_{n}+r_{n}N\left(1-\frac{N}{K_{n}}\right)\left(\frac{T_{i}}{T_{i}+m_{n}}\right)+\zeta \frac{CN}{C+h_{n}}-\delta_{n}\left(T_{u}+T_{i}\right)N-\gamma_{n}N \end{array}$$

(10) $$\begin{array}{ccc} \;T_{u}\left(0\right) & > & 0,\;T_{i}\left(0\right),\;V\left(0\right),X\left(0\right),\;Y\left(0\right),\;C\left(0\right),\;W\left(0\right),\;Z\left(0\right),N\left(0\right)\geq 0,\;T_{u}\left(0\right)+T_{i}\left(0\right)\leq K_{t}. \end{array}$$

It is clear that solutions of (1)–(9) exist and remain nonnegative on [0,∞) so that the model is biologically feasible. When there is no OVT, s=0, (1)–(9) always has a unique tumor-free equilibrium $E_{0}=\left(0,0,0,0,0,0,0,\bar{N}\right)$, where $\bar{N}=s_{n}/\gamma_{n}$. It can be shown that $E_{0}$ is locally asymptotically stable if $r_{u}<d_{u}\bar{N}$ and $r_{i}<a_{t}$. See Appendix A. This indicates that the tumor can be eradicated if it is small and has small growth rates.

### 2.2. Parameter Estimation

In this section, we estimate parameter values from literature.

**Susceptible tumor cells.** Susceptible tumor cells grow logistically with intrinsic growth rate $r_{u}=0.924$ day${}^{-1}$ and carrying capacity $K_{t}=3.3\times 10^{9}$ cells. These values are taken from [35]. An uninfected tumor cell becomes infected after infection from oncolytic viruses. The viral infection rate $\beta_{t}=0.0038$ (cells)(PFU${}^{-1}$)(day${}^{-1}$) and the half-saturation constant $m_{v}=1$ cells are adopted from [26]. An uninfected tumor cell can be killed by either effector cells or cytokines. The tumor killing rate $\delta_{c}=0.2$ (cells)(day${}^{-1}$)(pg/mL)${}^{-1}$ by cytokines with half-saturation constant $m_{t}=10^{5}$ cells are taken from [36]. The half-saturation constant for the killing by effector cells is $m_{x}=10^{3}$ cells. The lysis rate of tumor cells (infected and uninfected) by immune cells is $\delta_{x}=2$ day${}^{-1}$. Both values of $m_{x}$ and $\delta_{x}$ are from [26]. The tumor (infected and uninfected) killing rates due to NK cells $d_{u}$ and $d_{i}$ are the same with $8.68\times 10^{-10}$ (days${}^{-1}$)(cells${}^{-1}$) and taken from [15,17].

**Infected tumor cells.** Infected tumor cells grow logistically with intrinsic growth rate $r_{i}=0.924$ day${}^{-1}$ and carrying capacity $K_{t}=3.3\times 10^{9}$ cells. It has a disease related death rate $a_{t}$. The range of $a_{t}$ is 0.5−2.6667 (cell${}^{-1}$)(day${}^{-1}$) rescaled from [29]. We set $a_{t}=1$. This value is also adopted from [15] and [16]. In addition to the killings by CD8^+^ T cells and cytokines, infected tumor cells can be killed by anti-viral immune cells, and this lysis rate is $\delta_{z}=1$ (cell${}^{-1}$)(day${}^{-1}$) according to [15].

**Oncolytic virus.** An infected tumor cell can produce many new viruses after being lysed. The burst size of virus particles released per lysed infected tumor celll, $b_{t}=1949$ (PFU)(cell${}^{-1}$), follows from [37]. Viruses have a natural death rate $\gamma_{v}$. We adopt $\gamma_{v}=2.55$ day${}^{-1}$ as in [38]. Virus killing rate by anti-viral immune cells and NK cells is $\delta_{v}=2.4\times 10^{-4}$ (cell${}^{-1}$)(day${}^{-1}$) and $d_{v}=0.12$ (cell${}^{-1}$)(day${}^{-1}$), respectively, according to [29].

**Tumor-specific immune cells (CD8^+^ T cells).** Tumor cells activate CD8^+^ T cells with the rate $a_{x}=0.0375$ (cell${}^{-1}$)(day${}^{-1}$) taken from [15]. Range of the proliferation rate of anti-tumor immune cells is $2.4\times 10^{-4}$–2.4 (cell${}^{-1}$)(day${}^{-1}$) rescaled from [29] with $10^{-5}$–$10^{-1}$ per hour. The half-saturation constant, $h_{x}=40$ cells, is adopted from [15]. The death rate of anti-tumor immune cells is $\gamma_{x}=0.1$ day${}^{-1}$ taken from [26]. T cells are recruited by immune cells through interactions with the virus at a rate $a_{v}=2\times 10^{-6}$ (PFU${}^{-1}$)(day${}^{-1}$). This is a rough estimate since this relationship has not been studied previously.

**Anti-tumor immune cells (CD4^+^ T cells).** CD4^+^ T cells can kill cancer cells with cytokines. The half-saturation constant for tumor cell population detected by T cells, $h_{y}=10^{3}$ cells, proliferation rate of Th cells, $a_{y}=0.09$ (cells)(days${}^{-1}$)(pg/mL)${}^{-1}$, and apoptosis rate of Th cells, $\gamma_{y}=0.1$ (day${}^{-1}$), have been taken from [27]. The measure of CTLA-4-mediated inhibition on CD4^+^ T cells, $\nu =10^{-3}$ molecule${}^{-1}$ is also taken from [27]. Notice that the feasible range of half-saturation constant is from 40 to $10^{5}$ cells in [29].

**Tumor-suppressive cytokines (IFN-γ).** Both CD8 and CD4 Th1 effector T cells are the primary sources of IFN-γ. CD8^+^ T cells produce copious amounts of IFN-γ in response to activation. The IFN-γ production in CD8^+^ T cells is central to the generation of Th1 immune responses through NFAT1 protein [39]. The IFN-γ production rate by CD8^+^T cells is $\alpha_{x}=9$ (pg/mL)(day${}^{-1}$)(cell${}^{-1}$)(cell${}^{-1}$). The IFN-γ production rate by CD4^+^ T cells is $\alpha_{y}=9$ (pg/mL)(day${}^{-1}$)(cell${}^{-1}$)(cell${}^{-1}$), while the loss rate of tumor-suppressing cytokines is $\gamma_{c}=34$ day${}^{-1}$. These values follow from [27,40]. NK cells are also the major source of IFN-γ. The IFN-γ production rate by NK cells is estimated as $\alpha_{n}=0.4$ (pg/mL)(day${}^{-1}$)(cell${}^{-1}$)(cell${}^{-1}$). For different subjects, the parameter range for the measure of inhibition varies from $10^{-3}$ to 1. We estimate the measure of inhibition of CTLA-4 to CD8^+^ T ($b_{x}$), CD4^+^ T cells ($b_{y}$), and NK cells ($b_{n}$) based on the model validation. The measure $b_{x}$ of CTLA-4-mediated inhibition on IFN-γ produced by CD8^+^ T cells has the value $b_{x}=10^{-3}$ molecule${}^{-1}$. The measure $b_{y}$ by CD4^+^ T cells has $b_{y}=10^{-3}$ molecule${}^{-1}$, and the measure $b_{n}$ by NK cells has $b_{n}=10^{-3}$ molecule${}^{-1}$.

**Immune checkpoint CTLA-4.** The CTLA-4 express rate on a single CD4^+^ T cell is assumed to be a constant with $r_{y}=5000$ (molecules)(day${}^{-1}$)(cell${}^{-1}$). The degradation rate of CTLA-4 has $\gamma_{w}=8.3178$ day${}^{-1}$. They are from [27]. It is estimated using the assumption of exponential decay for the degradation of CTLA-4. The CTLA-4 express rate on a CD4^+^ T cell is estimated in the range of 2500–5000 (molecules)(day${}^{-1}$)(cells${}^{-1}$) under the linear growth assumption. This estimation is due to the fact that the maximum number of CTLA-4 molecular is $10^{4}$ per cell and the maximal protein CTLA-4 expression is being reported at 48–96 hours post-activation in Jaffe [41]. The CTLA-4 express rate on a single CD8^+^ T cell is estimated as $r_{x}=800$ (molecules)(day${}^{-1}$)(cell${}^{-1}$). The estimation is based on the fact that the relative expression of CTLA-4 is higher in CD4^+^ T cells compared with CD8^+^ T cells [20]. The inhibition rate, *u* (dimensionless), of immune cells by checkpoint CTLA-4 is from 0 to 1.

**Virus-specific immune cells.** The main function of viral-specific immune cells is to kill viruses from the therapy. The birth rate of anti-viral immune cells in response to the presence of viral particles on the surface of infected cancer cells is estimated as $p_{v}=0.6$ day${}^{-1}$ with a death rate $\gamma_{z}=0.13296$ day${}^{-1}$. They are from [15]. The feasible range of the infected cell-mediated proliferation rate due to anti-viral immune response is 0.6–2.5 day${}^{-1}$ from [16].

**Natural killer cells.** The constant influx of NK cell is $s_{n}=3.2\times 10^{3}$(cells)(day${}^{-1}$), the half-saturation constant of infected tumor cells is $m_{n}=10^{4}$ cells, the recruitment rate of NK cells via ICD by infected cells is $r_{n}=10^{-5}$ day${}^{-1}$, the carrying capacity for NK cell population is given by $K_{n}=6.63\times 10^{10}$ cells, the inactivation rate of NK cells by tumor cells is $\delta_{n}=10^{-7}$ (cell${}^{-1}$)(day${}^{-1}$), and the natural death rate of NK cells is $\gamma_{n}=4.12\times 10^{-2}$ day${}^{-1}$. These values are adopted from [17]. The stimulation and recruitment rate of NK cells by IFN−γ is ζ=0.5 days${}^{-1}$ which is estimated from [42]. The half-saturation constant for NK cell population activated by cytokines is $h_{n}=3\times 10^{2}$ (pg/mL)${}^{-1}$ followed from [34].

Table 3 summarizes model parameter values and value ranges, and values used in simulations.

## 3. Numerical Simulations

Since the time period for majority of the experimental studies on tumor–immune system interactions are in the order of few days/weeks after initial injections of oncolytic virus therapy, our study of model (1)–(9) begins by investigating transient behavior of the system as we vary the dosages of immunotherapy and immune responses. We validate the model according to the experimental data of Engeland et al. [5]. In [5], tumor sizes in mice during combined therapy with anti-CTLA4 were recorded. In particular, malignant melanoma B16-CD20 cells were injected subcutaneously into the flank of C57BL/6 mice. When tumors had reached an average volume of 40 mm${}^{3}$ ($4\times 10^{7}$ number of cells), mice were treated with carrier fluid treatment (mock; control) or injected with $2\times 10^{6}$ viruses (OV) in the tumor, or injected with viral particles together with anti-CTLA-4 (OV-aCTLA-4) for 5 consecutive days [5]. They revealed that the combination therapy had a synergistic effect, which enhances host anti-tumor immunity and increases the efficacy of OV treatment alone. Evolutions of tumor volumes for three different treatments on day 18 after implantation are shown in Figure 2c of Engeland et al. [5]. On day 18, the tumor volume reached 1860 mm${}^{3}$ ($1.86\times 10^{9}$ number of cells), 1140 mm${}^{3}$ ($1.14\times 10^{9}$ number of cells), and 400 mm${}^{3}$ ($4\times 10^{8}$ number of cells) approximately for mice treated with mock, OVs, and OV-aCTLA-4, respectively. The tumor volumes for mice treated with OV-aCTLA-4 are significantly lower than the controlled mice of no treatment. In our numerical simulations, tumor volumes are translated to the number of tumor cells through the relation of 1 cm${}^{3}$ = $10^{9}$ number of cells [46]. The simulations are performed by MATLAB ode15s ODE solver. Treatments with 5 consecutive days are represented by a continuous function. The treatment of oncolytic virus therapy is represented by the parameter *s*, and the blocking rate of CTLA-4 is represented by the parameter *u*.

### 3.1. Model Validation

We start our numerical investigation on the dynamics of the model given in Section 2.1.1 by showing that our model output is in reasonable agreement with the data given in Figure 2c of Engeland et al. [5]. For preliminary mathematical analysis of the model, see Appendix A. According to the mice experiment of Engeland et al. [5], the treatment started on day 8 post-implantation and the tumor reached an average number of $4\times 10^{7}$ cells. Therefore, the initial condition for the susceptible tumor is $T_{u}\left(0\right)=4\times 10^{7}$. Following the injection of tumor cells, it is reasonable to assume that the tumor activated primary immune responses. The primary immune responses are represented by the initial number of CD8^+^ T cells, CD4^+^ T cells, and NK cells. For simplicity, the initial settings of anti-tumor immune cells of CD8^+^ T and CD4^+^ T cells are assumed to be the same with X(0)=Y(0)=250. All implementations last for 5 consecutive days. Initial settings of other variables are $T_{i}\left(0\right)=V\left(0\right)=C\left(0\right)=W\left(0\right)=Z\left(0\right)=0$ and $N\left(0\right)=10^{4}$ from [17].

**Figure 2:**
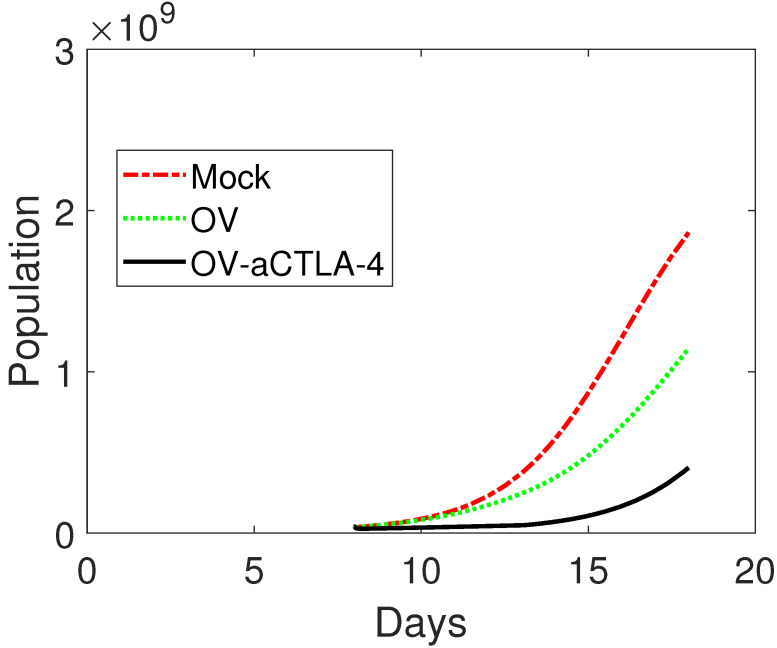
Comparison among numerically simulated dynamics of susceptible tumor cells for MOCK, OV, and OV-aCTLA-4 after treatments. The initial condition is $\left(4\times 10^{7},0,0,250,250,0,0,10^{4}\right)$. The treatment is administered on day 8 as the tumor reaches an average volume of $4\times 10^{7}$ cells and continues for 5 days. For OV only, the doses of oncolytic virus are $s=2\times 10^{6}$. The same amount of oncolytic virus is applied to OV-aCTLA-4 treatment with 0.76 blockade rate of CTLA-4. Other parameter values are listed in Table 3.

Validation starts from the case of no treatment. Our simulation results show that the tumor grows up to $1.8630\times 10^{9}$ on day 18 without any treatment. Without any treatment, the tumor size is approximately $1.8\times 10^{9}$ in Engeland et al. on day 18 [5]. For treatment with OV alone, our numerical simulation shows that, if $2\times 10^{6}$ oncolytic viruses are injected into the mice when the tumor reaches an average volume of $4\times 10^{7}$ cells, the tumor reaches $1.1441\times 10^{9}$ cells on day 18 after 5 days of treatment on day 8 as shown in Figure 2. Notice that the average tumor size is approximately $1.14\times 10^{9}$ cells on day 18 in Engeland et al. if monotherapy OV is administered into mice [5]. For treatment with OV-aCTLA-4, if $2\times 10^{6}$ oncolytic viruses together with 0.76 rate of blockade of CTLA-4 are implemented on day 8 post-implantation and holds for 5 days, the tumor size is $4.0543\times 10^{8}$ cells on day 18. See Figure 2. Note that the tumor size is around $4\times 10^{8}$ cells on day 18 in Engeland et al. if mice are treated with combination therapy [5]. From Engeland et al. and our simulation results, tumor volumes on day 15 after implantation revealed a significantly lower tumor volume in mice treated with OV-aCTLA-4 compared to mock. It reveals that treatment with OV led to a delay in tumor progression. However, reduced tumor volumes at early time points did not prolong overall survival in mice treated with OV-aCTLA-4 from Engeland et al. [5]. From Figure 2e of Engeland et al., the survival rate is zero or close to zero for mice treated with OV-aCTLA-4 on day 30 post-implantation. Our simulation results also show that, with OV-aCTLA-4, the tumor will grow to $2.5746\times 10^{9}$ cells on day 30 post-implantation.

### 3.2. Treatment Protocol for Improved Therapeutic Outcomes

**Figure 3:**
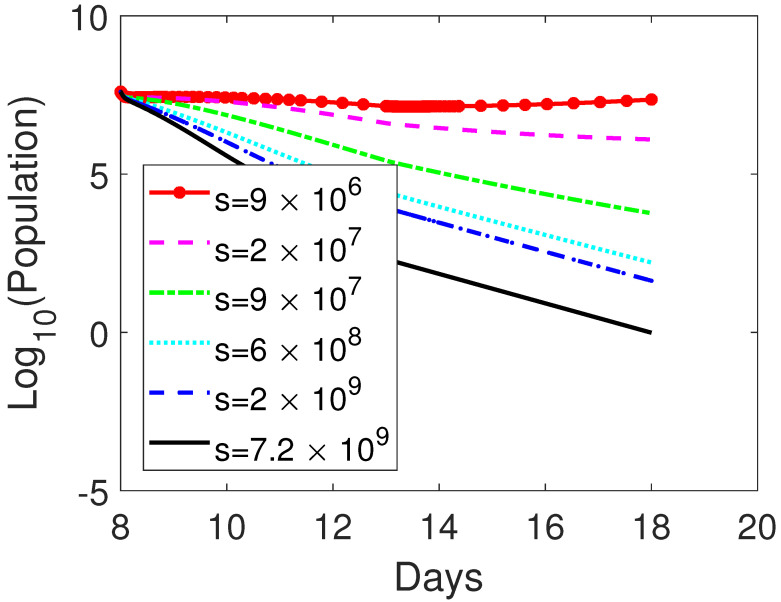
Comparison among numerically simulated dynamics of susceptible tumor cells for OV-aCTLA-4 treatment on day 18 post-implementation with various amounts of oncolytic virus. The initial condition is as in Figure 2. The level of oncolytic virus is represented by *s* as indicated in the figure. The treatment started on day 8 post-implantation as the tumor size reaches $4\times 10^{7}$ and lasted for 5 days. The same CTLA-4 blocking rate as in Figure 2, u=0.76, is applied to OV-aCTLA-4 treatment. Other parameter values are listed in Table 3.

In the above experiment, none of the treatment protocol can suppress the tumor completely on day 18. We continue to investigate whether tumors can be completely killed (the number of tumor cells is less than one) on day 18 post implantation with the same treatment schedule but various dosages. We either vary the amount of oncolytic virus or the blockade rate of CTLA-4 or both. With the same initial settings and obstructing rate of CTLA-4, u=0.76, if the dosage of oncolytic virus is $s=9\times 10^{6}$ on day 8 as the tumor size reaches to $4\times 10^{7}$ and the treatment lasts for 5 days, the susceptible tumor size is $2.2660\times 10^{7}$ on day 18 post-implantation. If the dosage of oncolytic virus increases to $s=2\times 10^{7}$, the susceptible tumor size is reduced to $1.2457\times 10^{6}$. As the dosage of oncolytic virus further increases to $9\times 10^{7}$, $6\times 10^{8}$, and $2\times 10^{9}$, the tumor size is $5.8229\times 10^{3}$, 160.9773, and 42.7871, respectively. The tumor can be eradicated ($T_{u}=0.9755$) on day 18 if $s=7.2\times 10^{9}$. See Figure 3. Note that the amount of oncolytic virus ranging from $10^{6}$ to $10^{9}$ is adopted in our numerical investigations. On the other hand, $10^{9}$ is used in [15] and $10^{7}$ to $10^{8}$ is implemented in [16], whereas $2\times 10^{6}$ is infused in the mouse experiment of Engeland et al. [5]. The virus inoculum is often manipulated in clinical trials in the orders of magnitude ($10^{3}$–$10^{10}$) [15]. Thus, the level of oncolytic virus is safe in our simulation.

**Figure 4:**
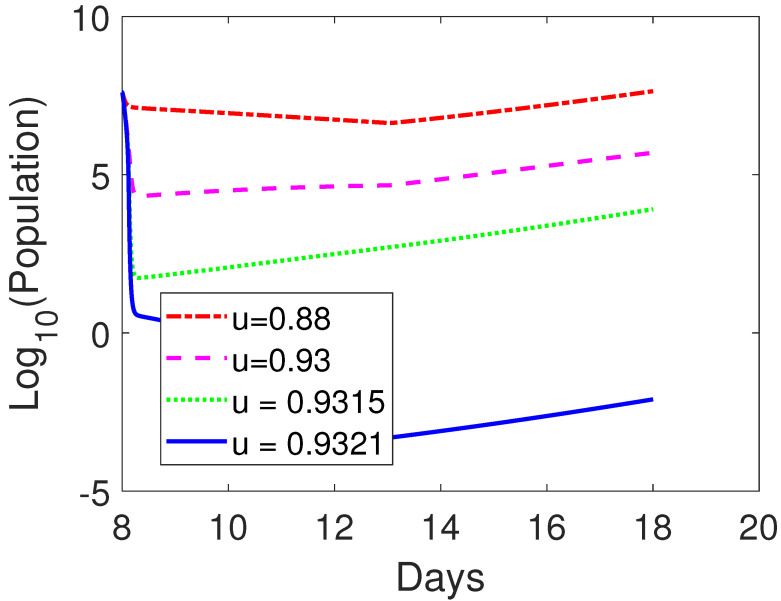
Comparison among numerically simulated dynamics of susceptible tumor cells for OV-aCTLA-4 treatment with various blockade rates of CTLA-4 on day 18 post-implementation. The initial condition is as in Figure 2. The various blockade rate of CTLA-4 is represented by *u* as in the figure. The therapy is administered on day 8 and holds for 5 days. The same dosages of oncolytic virus as in Figure 2, $s=2\times 10^{6}$, is applied to OV-aCTLA-4 treatment. Other parameter values are given in Table 3.

Next, we examine the influences of the blocking rate of CTLA-4 to the tumor size on day 18 by fixing the amount of oncolytic virus at $s=2\times 10^{6}$ and only varying the CTLA-4 blocking rate *u*. When the CTLA-4 blocking rate is varied between u=0.93 and u=0.9315, the corresponding tumor size is $4.9361\times 10^{5}$ and $8.1300\times 10^{3}$, respectively. However, if u=0.9321, the tumor size dropped to $T_{u}=0.0080$ on day 18. The tumor can be eradicated on day 18. See Figure 4. It shows that the CTLA-4 blocking rate is very sensitive when it is above a threshold and there exists a critical CTLA-4 blocking rate for tumor eradication.

Following from the above results, the level of oncolytic virus and the rate of blockade of CTLA-4 have deterministic influences on the treatment outcomes. With the above suggested treatment protocols, either the therapeutic outcomes can be improved or the tumor can be eradicated on day 18 after implantation. Observe that the level of oncolytic virus or the CTLA-4 blocking rate has to be high to eradicate the tumor on day 18. The high level of dosages may result in immense immunopathology and cause immune-related adverse events. Therefore, we continue to explore the possible treatment protocol of effective therapy with reduced dosages. If u=0.87, and $s=5\times 10^{9}$, the tumor size is 0.2893 on day 18. If u=0.818, and $s=6\times 10^{9}$, the tumor size is 0.9669 on day 18. If u=0.77, and $s=7\times 10^{9}$, the tumor size is 0.8133 on day 18. The tumor is possible to be suppressed on day 18 if the above treatment protocols are administered following from our numerical results.

### 3.3. Parameter Sensitivity Analysis

We start by performing global sensitivity analysis of mono-therapy of OVT and also of combined treatment of OVT and anti-CTLA-4 to replicate in silico of a virtual experimental trial with 500 different mice. Sensitivity analysis is used to identify primary parameters that influence treatment efficacy. The majority of range of parameter values are estimated based on one over ten to twice the baseline values as in [27], and otherwise using estimates in the literature when available. The range of values of each model parameter is shown in Table 3. We perform sensitivity analysis using partial rank correlation coefficient (PRCC) analysis and Latin hypercube sampling (LHS) [47,48]. The sensitivity indices of the PRCC vary between –1 and +1, which measure nonlinearly but the strength of monotonic relation between output variables and parameters of interest. The global sensitivity analysis is obtained at the endpoint t=10 days. This, in particular, is correspondent to 18 days post-implantation, in agreement with the model validation. Note that the dummy parameter does not belong to the model parameters. The model parameters with sensitivity indices less than or equal to that of the dummy parameter are considered not significantly different from zero [47].

#### 3.3.1. Monotherapy of Oncolytic Virus

**Figure 5:**
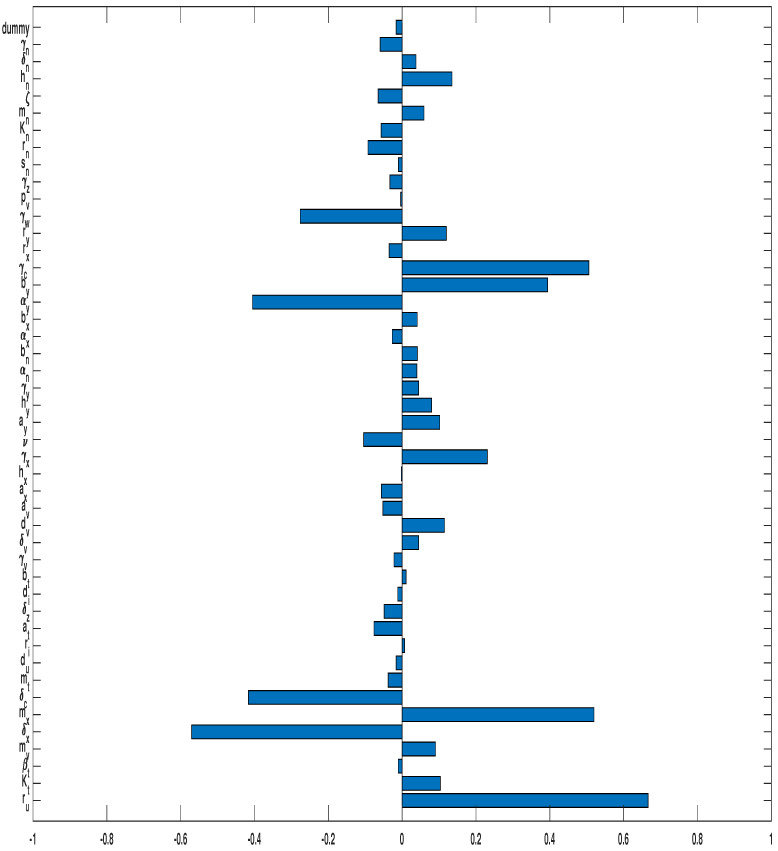
PRCC of susceptible tumor size when the sole OVT is applied.

In this subsection, we investigate the model results when the immune checkpoint inhibitor is not applied. Figure 5 shows the PRCC analysis result in the scenario where each parameter in the model is considered. For this case, the parameters with the strong correlation to the susceptible tumor size are tumor growth rate $r_{u}$, with $P(r_{u})=0.6663$, tumor killing rate by immune cells $\delta_{x}$, with $P(\delta_{x})=-0.5705$, half-saturation constant of the tumor killing rate by effector cells $m_{x}$, with $P(m_{x})=0.5195$, decay rate of tumor-suppressing cytokines $\gamma_{c}$, with $P(\gamma_{c})=0.5057$, and tumor killing rate by cytokines $\delta_{c}$, with $P(\delta_{c})=-0.4167$. See Figure 5.

#### 3.3.2. Combined OVT with Anti-CTLA4

**Figure 6:**
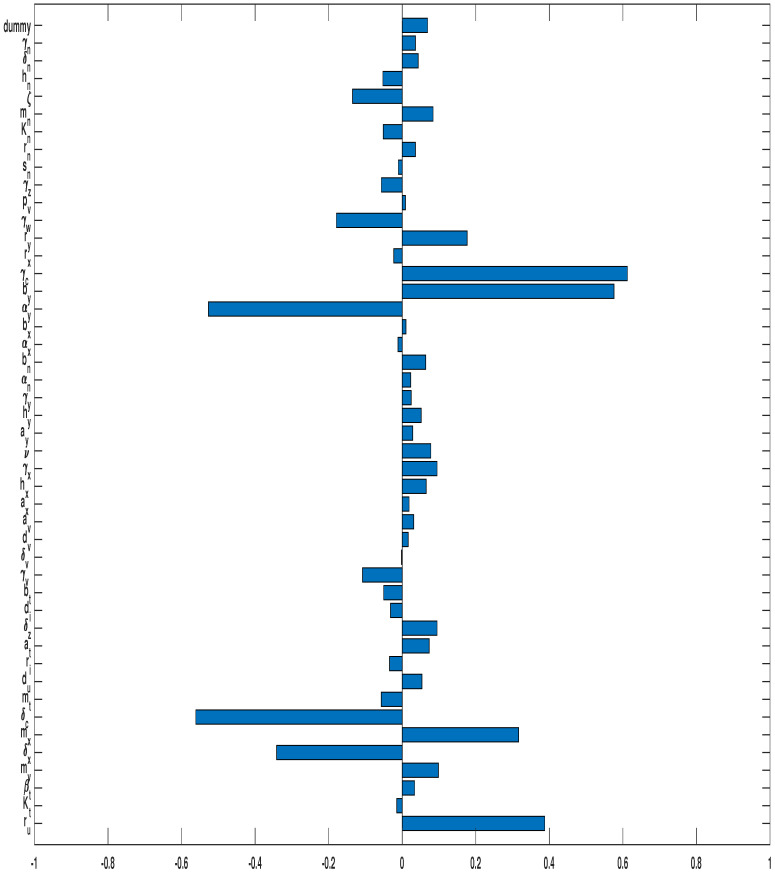
PRCC for the oncolytic virus parameters with anti-CTLA-4 against susceptible tumor cells. In all simulations, the tumor was treated with OVT and anti-CTLA-4.

We now discuss the model results when combined OVT and the immune checkpoint inhibitor, anti-CTLA-4, are administered. Figure 6 shows the results of PRCC for each parameter in the model. We denote this PRCC by $\hat{P}$ for the combined treatment scenarios. For this case, the parameters have a strong relationship with the susceptible tumor size are decay rate of tumor-suppressing cytokines $\gamma_{c}$, with $\hat{P}\left(\gamma_{c}\right)=0.6121$, measure of CTLA-4-mediated inhibition on IFN-γ of CD4^+^ T cells $b_{y}$, with $\hat{P}\left(b_{y}\right)=0.5760$, tumor killing rate by cytokines $\delta_{c}$, with $\hat{P}\left(\delta_{c}\right)=-0.5613$, IFN-γ production rate of CD4^+^ T cells $\alpha_{y}$, with $\hat{P}\left(\alpha_{y}\right)=-0.5270$, and tumor growth rate $r_{u}$, with $\hat{P}\left(r_{u}\right)=0.3875$.

**Figure 7:**
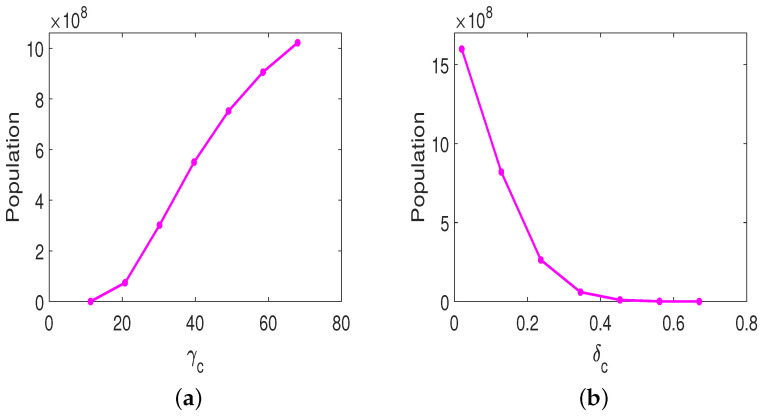
Susceptible tumor size with combination therapy. The tumor size is plotted against (**a**) $\gamma_{c}$, the decay rate of tumor-suppressing cytokines; (**b**) $\delta_{c}$, the tumor killing rate by cytokines.

Following from the above analysis, parameters $\gamma_{c}$, $r_{u}$, and $\delta_{c}$ play important roles on both the sole OVT and the combined therapy with anti-CTLA-4. We are interested in how important parameters $\gamma_{c}$ and $\delta_{c}$ influence the susceptible tumor size by the day 18 post-implantation, if the same treatment protocol as in Figure 2 is applied. Figure 7a shows susceptible tumor size against $\gamma_{c}$, the decay rate of tumor-suppressing cytokines. If we increase $\gamma_{c}$ to twice the baseline value ($\gamma_{c}=68$), the tumor size increases to $1.0220\times 10^{9}$ by day 18. The tumor size is approximately 2.5 times larger than the tumor size when $\gamma_{c}$ is at the baseline value. If we decrease $\gamma_{c}$ to one third value in Table 3, $\gamma_{c}=11.33$, the susceptible tumor size is close to zero on day 18. The tumor can be eradicated on day 18. Figure 7b provides susceptible tumor size against $\delta_{c}$, the tumor killing rate by cytokines. If we decrease $\delta_{c}$ to one tenth of the value in Table 3, $\delta_{c}=0.02,$ the susceptible tumor size increases to $1.5989\times 10^{9}$ on day 18 post-implantation. The tumor size is approximately 4 times larger than the tumor size when $\delta_{c}$ is at the baseline value. If we we increase $\delta_{c}$ to 0.67, the tumor size is $5.0596\times 10^{-6}$. The tumor can be eradicated on day 18 post-implantation. Collectively, parameters related to CD8^+^ T cell killing play significant roles for OVT only therapy, whereas parameters related to the level of IFN-γ secreted by CD4^+^ T cells are important for combined OVT with anti-CTLA4. In addition, the tumor growth rate, decay rate of tumor-suppressing cytokines, and tumor killing rate by cytokines are important factors influencing the behavior and fate of the tumor.

To pinpoint the parameters that have a major effect on the combined therapy in relation to the sensitivity analysis of monotherapy of OVT, we investigate the rate of PRCCs between the analysis with sole OVT and the analysis of combined therapy. Among significant parameters, the most marked distinction between this part of analysis with the previous analysis of monotherapy of OVT is the parameter $b_{y}$, which is the measure of CTLA-4-mediated inhibition on IFN-γ produced by CD4^+^ T cells. With anti-CTLA-4, the PRCC between $b_{y}$ and tumor size is $\hat{P}\left(b_{y}\right)=0.5760$, while it is $P(b_{y})=0.3941$ when only OVT is applied. Thus, parameter $b_{y}$ exhibits a much stronger correlation with the susceptible tumor size after treatment when the tumor is administered with additional anti-CTLA-4. This indicates that the measure of CTLA-4-mediated inhibition on IFN-γ produced by CD4^+^ T cells gives more significance to the efficacy of the combined treatment than to the effectiveness of the sole OV therapy. The second and third marked distinction between current analysis and the result with sole OVT are related to the parameters $\delta_{c}$, the tumor killing rate by cytokines, and $\alpha_{y}$, the IFN-γ proliferation rate by CD4^+^ T cells, respectively. With anti-CTLA-4, the PRCC values of $\delta_{c}$ and $\alpha_{y}$, with respect to the tumor size are $\hat{P}\left(\delta_{c}\right)=-0.5613$, and $\hat{P}\left(\alpha_{y}\right)=-0.5270$, respectively, while with single dose of OVT, the corresponding PRCC values are $P(\delta_{c})=-0.4167$, and $P(\alpha_{y})=-0.4054$, respectively. It is suggesting that the amount of IFN-γ secreted by CD4^+^ T cells contributes more significantly to the cogency of the combined treatment than to the effectiveness of sole OVT. We also note that the tumor growth rate, $r_{u}$, the lysis rate of tumor cells by immune cells, $\delta_{x}$, and the half-saturation constant of cytotoxic killing rate by immune cells, $m_{x}$, are much less significant with combined therapy than with the monotherapy OVT. It is re-emphasized that IFN-γ plays a more important role on eradicating tumor cells with combined immunotherapies.

**Figure 8:**
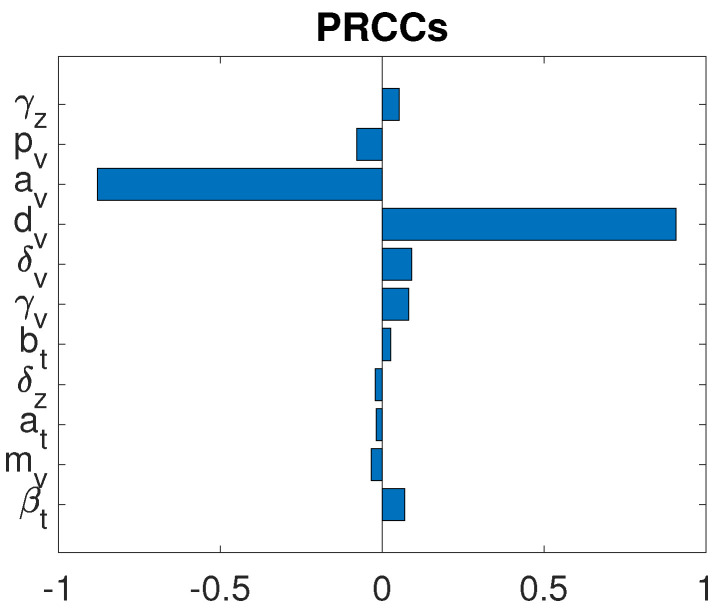
GSA for the oncolytic virus related parameters under combination therapy.

**Figure 9:**
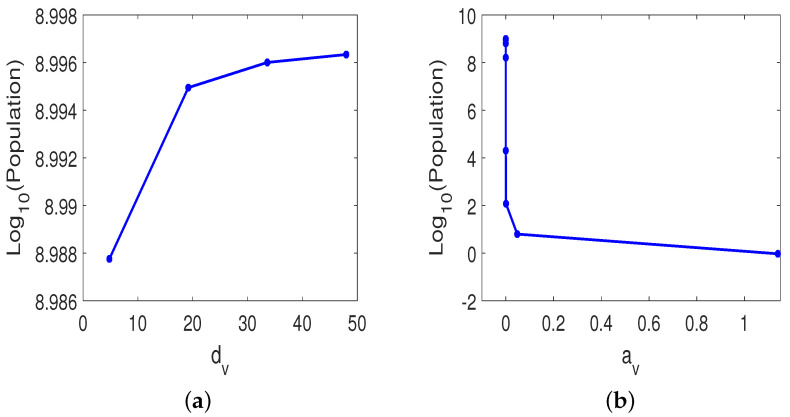
Susceptible tumor size in oncolytic virus sensitivity analysis. The tumor size is plotted against (**a**) $d_{v}$, the killing rate of virions by innate immune cells; (**b**) $a_{v}$, the proliferation rate of immune cells induced by oncolytic virus.

Particularly, we are interested in the role of oncolytic viruses on the treatment success. To study this effect, we implement different global sensitivity analysis by varying only those parameters associated with the OVs while keeping all other parameters fixed. This simulates an experiment of mice with comparable similar tumors and immune response but treated with various characters of viruses. We realized that the most two significant oncolytic virus-related parameters are the killing rate $d_{v}$ of virions by innate immune cells, and the proliferation rate $a_{v}$ of immune cells due to OVs. Figure 8 shows that the PRCCs for $d_{v}$ and $a_{v}$ were $\hat{P}\left(d_{v}\right)=0.9074$ and $\hat{P}\left(a_{v}\right)=-0.8801$, respectively. It indicates the strong correlation between these two parameter values and the post-treatment susceptible tumor cell population. The left plot in Figure 9a illustrates the tumor size as a function of killing rate of virions by innate immune cells, $d_{v}$. With the same treatment protocols used in Figure 2, but if $d_{v}$ is increased to 4.8, 19.2, or 33.6, the susceptible tumor size gradually increases to $9.7220\times 10^{8}$, $9.8844\times 10^{8}$, or $9.9085\times 10^{9}$, respectively on day 18. If $d_{v}$ reaches the upper feasible value, 48, in Table 3, the susceptible tumor size grows up to $9.9161\times 10^{8}$. This strong correlation reflects the fact that host immune resistance is a major obstacle to intravenous delivery of OVs. Particularly, NK cells are known to indiscriminately attack both uninfected and OV-infected tumor cells rapidly [33,49]. This rapid clearance, however, may halt the desired spread of OVs and hence diminish overall OVT efficacy. Consequently, NK cell response should be minimized in order to allow viruses to replicate sufficiently [50,51]. This result is consistent with preclinical and clinical studies [17].

The right panel in Figure 9b plots tumor size against immune cell proliferation rate $a_{v}$ activated by oncolytic viruses. The simulation endpoint is on t=18 day. As $a_{v}$ decreases respectively to $4\times 10^{-6}$, $10^{-6}$, $2\times 10^{-7}$, or $4\times 10^{-9}$, the tumor size increases to $1.6255\times 10^{8}$, $6.4039\times 10^{8}$, $9.1278\times 10^{8}$, or $9.9287\times 10^{8}$, respectively. On the other hand, if $a_{v}$ increases respectively to $6\times 10^{-5}$, $8\times 10^{-4}$, 0.048, or 1.14, the tumor size will reduce to $2.0135\times 10^{4}$, 118.5440, 6.2881, or 0.9339, respectively. The importance of parameter $a_{v}$ reveals that the major role of the combined therapy is its activation of host anti-tumor immune system responses to post-treatment susceptible tumor population rather than to its direct destruction of the tumor cells.

## 4. Discussion

In this work, we developed a mathematical model to investigate the treatment of murine model of malignant melanoma B16-CD20 using an immune checkpoint inhibitor anti-CTLA-4 and OVT. The model is constructed to describe the interactions between the tumor populations (susceptible and infected), virus population, tumor-specific CTL response, natural killer cell populations, virus-specific immune populations, tumor suppression cytokines, and the effect of immune checkpoint inhibitor anti-CTLA-4. In particular, we considered the effects of both types of effector cells, namely CD8^+^ T and CD4^+^ T cells. In our model, CD8^+^ T cells play a direct role of tumor killing, whereas CD4^+^ T cells kill cancer cells through cytokines they produced. T cells are also recruited by immune cells which become activated when they encounter the oncolytic viruses. Another group of immune cells we considered in the model is NK cells. They can be specific or non-specific during oncolytic virotherapy. NK cells become activated due to immunogenic cell death of infected tumor cells and interferons. They kill tumor cells and clear free viruses. Cytokines activated by both susceptible and infected tumor cells, and secreted by activated T cells and NK cells, conduct the indirect killing. CTLA-4 molecules are expressed on CD8^+^ T and CD4^+^ T cells and can prevent the cytokine production. Fragments from infected cancer cells activate the anti-viral immunity which subsequently kills infected cells and clear free virus. Our study focused on the transient behavior of the system because the tumor-immune dynamics are displayed only within few days/weeks after the initial injection of OVs in most of the pre-clinic studies as well as in the mouse experiment. Our model was supported by the experimental data of Engeland et al. [5], which showed that mice treated with combined therapy would greatly reduce tumor burden comparing with the ones treated with either mock or only OVT. We took a step further to investigate treatment protocols to improve therapeutic outcomes by studying various amounts of oncolytic virus and rates of blockade of CTLA-4. It was found that the tumor burden can either be reduced or completely eradicated on day 18 post-implementation for the combined therapy if either the level of oncolytic virus or the rate of blockade of CTLA-4 is increased. The blockade rate of CTLA-4 is very sensitive when it was above a threshold and there exists a critical blockade rate of CTLA-4 for tumor eradication. Moreover, to avoid immense immunopathology due to high level of dosages, we also provide suggestions on effective treatment protocols with reduced dosages.

We performed sensitivity analyses with OVT alone and also with the combined therapy of OVT and anti-CTLA-4 to determine parameters that are most significantly impacting the tumor response to treatments. Our study revealed that parameters related to CD8^+^ T cells killings have a large impact on the treatment outcome with OVT alone, whereas parameters related to IFN-γ secreted by CD4^+^ T cells strongly influence treatment responses for combined treatment with anti-CTLA-4. In addition, tumor growth rate, decay rate of tumor-suppressing cytokines, and tumor killing rate by cytokines are important factors in influencing the behavior and fate of the tumor treatment. We also observed that the most three substantial differences between analysis with combined OVT and anti-CTLA-4 and the analysis with monotherapy of OVT are the measure $b_{y}$ of CTLA-4-mediated inhibition on IFN-γ of CD4^+^ T cells, the tumor killing rate $\delta_{c}$ by cytokines, and the IFN-γ production rate $\alpha_{y}$ by CD4^+^ T cells. It suggests that the IFN-γ produced by CD4^+^ T cells associated with immunity takes part in a much more important role in responding to the auxiliary therapy than to the efficacy of sole OVT. In addition, we performed sensitivity analyses only for parameters directly related to the oncolytic virus. One of the significant OV related parameters is the killing rate $d_{v}$ of virions by innate immune cells, reflecting the fact that host immune resistance is a major obstacle to intravenous delivery of OVs. It indicated that the virus must first survive interactions with antibody neutralization elimination effect in the blood circulatory system [52]. Immunosuppression inhibition such as anti-CTLA-4 can enhance the oncolytic virus therapy. The other significant oncolytic virus related parameter is the proliferation rate $a_{v}$ of immune cells evoked by the interactions with oncolytic virus, suggesting that the principal role of the combined therapy is its activation of host anti-tumor immune system responses to post-treatment susceptible tumor population rather than to its direct destruction of the tumor cells. Similar conclusions can be found in Storey et al. [29]. With combination therapy, the OVs infect susceptible tumor cells, whereas the lifespan of infected tumor cells is much shorter comparing to susceptible tumor cells. The anti-CTLA-4, on the other hand, stimulates IFN-γ production, which in turn kills tumor cells more effectively. Therefore, it is the combined effect of OVs and anti-tumor immunity that facilitates tumor destruction.

Despite the model’s ability on explaining how anti-CTLA-4 can improve virotherapy, we acknowledge that the model has some limitations. Our model is based on the population level interactions and does not account for subcellular events and stimulatory pathways. For example, the effect of IFN-γ on tumor gene expression [53], TLR-ligands derived from the OLVs [54], and T cell exhaustion [55] are not incorporated. Despite these limitations, the model outputs using plausible parameter values agree well with the experimental data of England et al.. Our mathematical model provides useful insights on the dynamics of OV-anti-CTLA4. More complex dynamic interactions between OV, tumor cells, CTLA-4 and other types of cytokines and immune responses will be considered in our future research. In addition, the doses and schedule of the treatment in the experiment may not be optimal. The optimal therapeutic protocol will be investigated for the combination therapy to determine how the schedule and dose can produce optimal treatment outcomes in future research.

## Figures and Tables

**Table 1:** Model variables.

Variable	Description
$T_{u}\left(t\right)$	total number of susceptible (uninfected) tumor cell population
$T_{i}\left(t\right)$	total number of infected tumor cell population
V(t)	total number of oncolytic virions
X(t)	total number of anti-tumor immune cells (CD8^+^ T cells)
Y(t)	total number of anti-tumor immune cells (CD4^+^ T cells)
C(t)	concentration of IFN-γ (pg/mL)
W(t)	total number of CLTA-4 protein population
Z(t)	total number of anti-viral immune cells
N(t)	total number of natural killer cells

**Table 2:** Parameter description.

Parameter	Description
$r_{u}$	Uninfected tumor growth rate
$K_{t}$	Tumor carrying capacity
$\beta_{t}$	Infection rate of tumor cells by the oncolytic virus
$m_{v}$	Half-saturation constant for the tumor cells infection
$\delta_{c}$	Tumor killing rate by cytokines
$m_{t}$	Half-saturation constant for the tumor cells killed by the immune cells and cytokines
$\delta_{x}$	Lysis rate of tumor cells (infected and uninfected) by immune cells
$m_{x}$	Half-saturation constant of cytotoxic killing rate by immune cells
$a_{t}$	Death rate of infected tumor cells
$\delta_{z}$	Lysis rate of the infected tumor cells by virus-specific immune cells
$\gamma_{v}$	Viral clearance rate
$b_{t}$	Burst size from infected tumor cells lysed by the oncolytic virus
$\delta_{v}$	Virus killing rate by anti-viral immune cells
$a_{v}$	T cells recruited rate in response to immune cells through interactions with the virus
$a_{x}$	Anti-tumor adaptive immune cells proliferation rate
$h_{x}$	Half-saturation constant of tumor cells due to tumor antigens
$\gamma_{x}$	Death rate of effector cells
ν	The measure of CTLA-4 blocking rate on CD4^+^ T cells
$h_{y}$	Half-saturation constant for tumor cell population detected by T cells
$a_{y}$	Proliferation rate of Th cells
$\gamma_{y}$	Apoptosis rate of Th cells
$\alpha_{x}$	IFN-γ proliferation rate by CD8^+^ T cells
$\alpha_{y}$	IFN-γ proliferation rate by CD4^+^ T cells
$b_{n}$	The measure of CTLA-4-mediated inhibition on IFN-γ produced by NK cells
$b_{x}$	The measure of CTLA-4-mediated inhibition on IFN-γ produced by CD8^+^ T cells
$b_{y}$	The measure of CTLA-4-mediated inhibition on IFN-γ produced by CD4^+^ T cells
$\gamma_{c}$	Natural degradation rate of tumor-suppressing cytokines
$\gamma_{w}$	Death rate of CTLA-4
$p_{v}$	Proliferation rate of virus-specific immune cells in response to antigens
$\gamma_{z}$	Decay rate of the anti-viral immune cells
$s_{n}$	Constant influx of NK cells
$r_{n}$	Recruitment rate of NK cells via ICD by infected cells
$K_{n}$	Maximum capacity for NK cell production
$m_{n}$	Half-saturation constant of infected tumor cells
ζ	Stimulation and recruitment rate of NK cells by IFN−γ
$h_{n}$	Half-saturation constant for NK cell population activated by cytokines
$\delta_{n}$	Inactivation rate of NK cells by tumor cells
$\gamma_{n}$	Decay rate of NK cells
$r_{i}$	The growth rate of infected tumor cells.
$d_{u}$	Killing rate of uninfected tumor cells by NK cells
$d_{i}$	Killing rate of infected tumor cells by NK cells
$d_{v}$	Killing rate of virions by innate immune cells
$\alpha_{n}$	IFN-γ proliferation rate by NK cells
$r_{x}$	CTLA-4-expressing rate on a single CD8^+^ T cell
$r_{y}$	CTLA-4-expressing rate on a single CD4^+^ T cell

**Table 3:** Parameter baseline value.

Parameter	Baseline	Range	Units	Reference
$r_{u}$	0.924	[0.12–1.2]	day${}^{-1}$	[29,35]
$K_{t}$	$3.3\times 10^{9}$	$[10^{8}$–$9.7\times 10^{9}]$	cells	[35,43]
$\beta_{t}$	0.0038	0.0038	(cells)(PFU${}^{-1}$)(day${}^{-1}$)	[26]
$m_{v}$	1	1	cells	[26]
$\delta_{c}$	0.2	0.2	(cell)(day${}^{-1}$)(pg/mL)${}^{-1}$	[36]
$m_{t}$	$10^{5}$	$10^{5}$	cells	[36]
$\delta_{x}$	2	[0.0096–4.8]	day${}^{-1}$	[26,29]
$m_{x}$	$10^{3}$	$10^{3}$	cells	[26]
$d_{u}$	$8.68\times 10^{-10}$	$8.68\times 10^{-10}$	(day${}^{-1}$)(cells${}^{-1}$)	[15,17]
$r_{i}$	0.924	[0.12–1.2]	day${}^{-1}$	[44] or [45]
$a_{t}$	1	[0.5–2.6667]	(cell${}^{-1}$)(day${}^{-1}$)	[15,16,29]
$\delta_{z}$	1	[0.0096–4.8]	(cell${}^{-1}$)(day${}^{-1}$)	[15]
$d_{i}$	$8.68\times 10^{-10}$	$8.68\times 10^{-10}$	(day${}^{-1}$)(cells${}^{-1}$)	[15,17]
$b_{t}$	1949	[10–1949]	(PFU)(cell${}^{-1}$)	[37]
$\gamma_{v}$	2.55	[0.024–24]	(day${}^{-1}$)	[29,38]
$\delta_{v}$	$2.4\times 10^{-4}$	$[2.4\times 10^{-5}$–0.0240]	(cell${}^{-1}$)(day${}^{-1}$)	[29]
$d_{v}$	0.12	[0.024–48]	(cell${}^{-1}$)(day${}^{-1}$)	[29]
$a_{v}$	$2\times 10^{-6}$	$2\times 10^{-6}$	(PFU${}^{-1}$)(day${}^{-1}$)	Estimated
$a_{x}$	0.0375	$[2.4\times 10^{-4}$–2.4]	(cell${}^{-1}$)(day${}^{-1}$)	[15,29]
$h_{x}$	40	[40–$10^{5}]$	cells	[15,29]
$\gamma_{x}$	0.1	0.1	day${}^{-1}$	[26]
ν	$10^{-3}$	$10^{-3}$	molecule${}^{-1}$	[27]
$a_{y}$	0.09	$[2.4\times 10^{-4}$–2.4]	(cells)(days${}^{-1}$)(pg/mL)${}^{-1}$	[27,29]
$h_{y}$	$10^{3}$	[40–$10^{5}]$	cells	[27,29]
$\gamma_{y}$	0.1	0.1	day${}^{-1}$	[27]
$\alpha_{n}$	0.4	0.4	(pg/mL)(day${}^{-1}$)(cell${}^{-1}$)(cell${}^{-1}$)	Estimated
$b_{n}$	$10^{-3}$	$10^{-3}$	molecule${}^{-1}$	Estimated
$\alpha_{x}$	9	9	(pg/mL)(day${}^{-1}$)(cell${}^{-1}$)(cells${}^{-1}$)	[27,40]
$b_{x}$	$10^{-3}$	$10^{-3}$	molecule${}^{-1}$	[27,40]
$\alpha_{y}$	9	9	(pg/mL)(day${}^{-1}$)(cell${}^{-1}$)(cell${}^{-1}$)	[27,40]
$b_{y}$	$10^{-3}$	$10^{-3}$	molecule${}^{-1}$	[27,40]
$\gamma_{c}$	34	34	day${}^{-1}$	[27,40]
$r_{x}$	800	[400–800]	(molecules)(day${}^{-1}$)(cell${}^{-1}$)	Estimated
$r_{y}$	5000	[2500–5000]	(molecules)(day${}^{-1}$)(cell${}^{-1}$)	[27]
$\gamma_{w}$	8.3178	8.3178	day${}^{-1}$	[27]
$p_{v}$	0.6	[0.6–2.5]	day${}^{-1}$	[15,16]
$\gamma_{z}$	0.13296	0.13296	day${}^{-1}$	[15]
$s_{n}$	$3.2\times 10^{3}$	$[3.2\times 10^{3}$–$3.2\times 10^{4}]$	(cells)(day${}^{-1}$)	[17]
$r_{n}$	$10^{-5}$	$10^{-5}$	day${}^{-1}$	[17]
$K_{n}$	$6.63\times 10^{10}$	$6.63\times 10^{10}$	cells	[17]
$m_{n}$	$10^{4}$	$10^{4}$	cells	[17,29]
$\delta_{n}$	$10^{-7}$	$10^{-7}$	(cell${}^{-1}$)(day${}^{-1}$)	[15,17]
ζ	0.5	0.5	day${}^{-1}$	[42]
$h_{n}$	$3\times 10^{2}$	$3\times 10^{2}$	(pg/mL)${}^{-1}$	[34]
$\gamma_{n}$	$4.12\times 10^{-2}$	$4.12\times 10^{-2}$	day${}^{-1}$	[17]

## Appendix A

**Proposition** **A1.**
*Solutions of (1)–(9) exist and remain nonnegative on [0,∞).*

**Proof.** Let $X=(T_{u},T_{i},V,X,Y,C,W,Z,N)$ and denote system (1)–(9) by $X^{\prime }=F\left(X\right)$, where $F=(F_{1},F_{2},F_{3},F_{4},F_{5},F_{6},F_{7},F_{8},F_{9})$. Since each $F_{i}$ is locally Liptschitz on $R_{+}^{8}$, there exists a unique solution on $\left[0,t_{0}\right)$ for the initial value problem (1)–(9), where $t_{0}>0$ may depend on initial conditions. Since $F_{j}\left(X\right)\geq 0$ for any X with $X_{j}=0$, solutions remain nonnegative on the interval of existence. Because ${\left(T_{u}+T_{i}\right)}^{\prime }{|}_{T_{u}+T_{i}>K_{t}}<0$, $T_{u}\left(t\right)+T_{i}\left(t\right)\leq K_{t}$ on the interval of existence. In addition, $V^{\prime }\leq b_{t}a_{t}K_{t}+s-\gamma_{v}V$ implies V(t)≤M for some M>0. Therefore, it can be easily seen that $X^{\prime }\leq AX+b$, where

$$A=\left(\begin{array}{ccccccccc} r_{u} & 0 & 0 & 0 & 0 & 0 & 0 & 0 & 0\\ r_{i} & 0 & \beta_{t} & 0 & 0 & 0 & 0 & 0 & 0\\ 0 & b_{t}a_{i} & 0 & 0 & 0 & 0 & 0 & 0 & 0\\ 0 & 0 & 0 & 0 & 0 & 0 & 0 & 0 & a_{v}M\\ 0 & 0 & 0 & 0 & 0 & a_{y} & 0 & 0 & a_{v}M\\ V0 & 0 & 0 & \alpha_{x}K_{t} & \alpha_{y}K_{t} & 0 & 0 & 0 & \alpha_{n}K_{t}\\ 0 & 0 & 0 & r_{x}\left(1-u\right) & r_{y}\left(1-u\right) & 0 & 0 & 0 & 0\\ 0 & p_{t} & 0 & 0 & 0 & 0 & 0 & 0 & 0\\ 0 & 0 & 0 & 0 & 0 & 0 & 0 & 0 & r_{n}+\xi \end{array}\right)$$

and $b={\left(0,0,s,a_{x},0,0,0,0,s_{n}\right)}^{tr}$. Since solutions of $W^{\prime }=AW+b$ are defined on [0,∞), solutions of (1)–(9) are thus defined on [0,∞). □

Models (1)–(9) always have a tumor- and virus-free equilibrium $E_{0}=\left(0,0,0,0,0,0,0,0,\bar{N}\right)$, where $\bar{N}=s_{n}/\gamma_{n}$. The Jacobian matrix evaluated at $E_{0}$ is given by

$$\left(\begin{array}{ccccccccc} r_{u}-d_{u}\bar{N} & 0 & 0 & 0 & 0 & 0 & 0 & 0 & 0\\ 0 & r_{i}-a_{t} & 0 & 0 & 0 & 0 & 0 & 0 & 0\\ 0 & b_{t}a_{t} & -\gamma_{v}-d_{v}\bar{N} & 0 & 0 & 0 & 0 & 0 & 0\\ a_{x}/h_{x} & a_{x}/h_{x} & a_{v} & -\gamma_{x} & 0 & 0 & 0 & 0 & 0\\ a_{y}/h_{y} & a_{y}/h_{y} & a_{v}\bar{N} & 0 & -\gamma_{y} & 0 & 0 & 0 & 0\\ \alpha_{n}\bar{N} & 0 & 0 & 0 & 0 & -\gamma_{c} & 0 & 0 & 0\\ 0 & 0 & 0 & r_{x}\left(1-u\right) & r_{y}\left(1-u\right) & 0 & -\gamma_{w} & 0 & 0\\ 0 & p_{v} & 0 & 0 & 0 & 0 & 0 & -\gamma_{z} & 0\\ {}* & * & * & * & * & * & * & * & -\gamma_{n} \end{array}\right),$$

where *s are unimportant entries. It follows that $E_{0}$ is asymptotically stable if $r_{u}<d_{u}\bar{N}$ and $r_{i}<a_{t}$. If one of these or both inequalities is reversed, then $E_{0}$ is a saddle point.

**Proposition** **A2.**
*Let s=0 and $\gamma_{v}>b_{t}\beta_{t}$. Then, solutions of (1)–(9) satisfying $\lim_{t\to \infty }T_{i}\left(t\right)=0=\lim_{t\to \infty }V\left(t\right)$.*

**Proof.** Notice $T_{i}^{\prime }\left(t\right)\leq \beta_{t}V-a_{t}T_{i}$ and $V^{\prime }\left(t\right)\leq b_{t}a_{t}T_{i}-\gamma_{v}V$ for t≥0. Consider

(A1) $$\left(\begin{array}{c} x^{\prime }\\ y^{\prime } \end{array}\right)=\left(\begin{array}{cc} -a_{t} & \beta_{t}\\ b_{t}a_{t} & -\gamma_{v} \end{array}\right)\left(\begin{array}{c} x\\ y \end{array}\right),x\left(0\right)=T_{i}\left(0\right),y\left(0\right)=V\left(0\right).$$

Observe that (A1) is a linear, cooperative two-dimensional system, and solutions of (A1) converge to (0,0) by the assumption $\gamma_{v}>b_{t}\beta_{t}$. Since $T_{i}\left(t\right)\leq x\left(t\right)$ and V(t)≤y(t) for t≥0, the claim is proven. □
